# Characterization of Stress Responses in a *Drosophila* Model of Werner Syndrome

**DOI:** 10.3390/biom11121868

**Published:** 2021-12-12

**Authors:** Derek G. Epiney, Charlotte Salameh, Deirdre Cassidy, Luhan T. Zhou, Joshua Kruithof, Rolan Milutinović, Tomas S. Andreani, Aaron E. Schirmer, Elyse Bolterstein

**Affiliations:** 1 Department of Biology, Northeastern Illinois University, Chicago, IL 60625, USA; derek.epiney@northwestern.edu (D.G.E.); charlottesalameh@yahoo.com (C.S.); dmcassidy3@gmail.com (D.C.); tracy.zhou@northwestern.edu (L.T.Z.); Joshua.Kruithof@gmail.com (J.K.); r-milutinovic@neiu.edu (R.M.); a-schirmer@neiu.edu (A.E.S.); 2 Department of Neurobiology, Northwestern University, Evanston, IL 60208, USA; tomasandreani2019@u.northwestern.edu

**Keywords:** Werner syndrome, stress, *Drosophila*

## Abstract

As organisms age, their resistance to stress decreases while their risk of disease increases. This can be shown in patients with Werner syndrome (WS), which is a genetic disease characterized by accelerated aging along with increased risk of cancer and metabolic disease. WS is caused by mutations in *WRN*, a gene involved in DNA replication and repair. Recent research has shown that *WRN* mutations contribute to multiple hallmarks of aging including genomic instability, telomere attrition, and mitochondrial dysfunction. However, questions remain regarding the onset and effect of stress on early aging. We used a fly model of WS (*WRNexo^Δ^*) to investigate stress response during different life stages and found that stress sensitivity varies according to age and stressor. While larvae and young *WRNexo^Δ^* adults are not sensitive to exogenous oxidative stress, high antioxidant activity suggests high levels of endogenous oxidative stress. *WRNexo^Δ^* adults are sensitive to stress caused by elevated temperature and starvation suggesting abnormalities in energy storage and a possible link to metabolic dysfunction in WS patients. We also observed higher levels of sleep in aged *WRNexo^Δ^* adults suggesting an additional adaptive mechanism to protect against age-related stress. We suggest that stress response in *WRNexo^Δ^* is multifaceted and evokes a systemic physiological response to protect against cellular damage. These data further validate *WRNexo^Δ^* flies as a WS model with which to study mechanisms of early aging and provide a foundation for development of treatments for WS and similar diseases.

## 1. Introduction

Aging is the culmination of a complex network of physiological and genetic processes resulting in cellular decline. In 2013, Lopez-Ortin et al. categorized common aging processes into the nine hallmarks of aging, a hierarchical framework of causes of cellular damage (e.g., genomic instability, and telomere attrition), damage responses (i.e., dysregulated nutrient sensing, mitochondrial dysfunction, and cellular senescence), and phenotypic manifestations of unrepaired damage (i.e., stem cell exhaustion and altered intercellular communication) [1]. Kennedy et al., contributed a similar list to the aging field, the seven pillars of aging, which drew upon broader categories of decline in cellular processes (proteostasis, metabolism, stem cells and regeneration, macromolecule damage, epigenetics, inflammation, and adaptation to stress) to focus more specifically on the extension of human “healthspan” [2]. The conceptual overlap in each of these frameworks demonstrates that processes governing aging are interrelated and causally difficult to separate from each other.

One unifying concept in identifying causes of aging is a balance between stressors and stress responses [3] in that aging can be promoted by stress levels that exceed the capacity of stress response mechanisms. Cells are routinely exposed to aging-promoting stressors (e.g., chemical stress, thermal stress, and stress from UV exposure) and rely on protective mechanisms (e.g., DNA repair pathways, and heat shock proteins) to prevent macromolecule damage [4]. Oxidative stress, a condition caused by the imbalance between reactive oxygen species (ROS) and antioxidants that can neutralize them, occurs both extrinsically through chemical stress and intrinsically as byproducts of cellular metabolism [5]. While additional ROS in a state of oxidative stress can contribute to aging, ROS produced below a certain threshold are essential to stress and metabolic regulatory signaling pathways that maintain cellular homeostasis [1]. Therefore, small amounts of stress may produce a hormetic effect and promote cellular survival [1,5,6]. Because cellular responses to intrinsic and extrinsic stress are highly interrelated and can create additive impacts on aging, “multiplex” stress resistance is likely required for slowing of the aging process [4].

*Drosophila* provide an excellent model to study the synergistic interactions of stress and aging. *Drosophila* have long been used in aging research, largely due to their short lifespans (70–90 days), short generation time (10 days), and ease of genetic manipulation, allowing for rapid manifestation of age-related pathologies and their genetic causes [7]. Numerous high-throughput and reproducible assays have been developed to investigate changes in physiological behavior (e.g., locomotor activity, and responses to stimuli) in both larval and adult *Drosophila* [8] and have been used to determine changes in lifespan and behavioral responses to age-related stressors such as oxidative stress [9,10,11,12,13,14] and thermal stress [9,14,15,16]. Additionally, *Drosophila* contain 75% of human disease-causing genes [17] and has been used to create models of many age-related diseases such as Parkinson’s, Amyotrophic Lateral Sclerosis (ALS), and Huntington’s [17,18,19].

Our research uses mutant *Drosophila* to model Werner syndrome (WS), a rare autosomal recessive progeroid disease caused by mutations that results in loss of function of the DNA repair gene, *WRN*. WS is characterized by accelerated aging and high incidence of aging-related pathologies such as cancer, heart disease, and metabolic syndromes like type II diabetes, dyslipidemia, and fatty liver [20]. As a member of the RecQ family of helicases, WRN has essential roles in DNA replication, transcription, recombination, and repair [21]. Observations from WS cells and WRN-deficient models link WRN with the nine hallmarks of aging, most notably telomere attrition, mitochondrial dysfunction, and genomic instability [22]. WRN has two catalytic domains that function to maintain genomic stability: a 3′ to 5′ ATP-dependent helicase activity as well as 3′ to 5′ exonuclease activity [21]. Additionally, some of WRN’s roles in maintaining genomic stability can be attributed to non-catalytic functions [22,23,24]. Though WRN’s function in DNA repair is most prominently linked to its role in non-homologous end-joining (NHEJ) [25,26,27,28], it has also been shown to interact with various proteins in the Base Excision Repair (BER) pathway [29,30,31,32]. Because BER is largely responsible for repairing DNA damage caused by oxidative stress, WRN’s involvement in BER suggests that the balance of ROS to antioxidants may be key in WS pathology.

Our WS model flies are mutant in *WRNexo* (*WRNexo^Δ^*), which is homologous to the exonuclease portion of human WRN [33,34,35]. While WRNexo lacks a RecQ helicase domain, it has been shown to have functional similarity to human WRN [33,34,35,36,37], providing us with a unique opportunity to investigate the exonuclease-dependent functions of WRN in the absence of the helicase. *WRNexo^Δ^* flies have deficiencies in DNA repair [38] and display phenotypes consistent with accelerated aging observed in WS including shortened lifespan, increased tumor incidence, lower locomotor activity, and low larval body fat [37]. Here, we use our WS fly model to better understand the link between stress response and aging by describing their responses to common stressors (oxidative, thermal, and starvation). We also investigate sleep patterns in young and old *WRNexo^Δ^* and discuss how behavioral changes may demonstrate an adaptation to stress. This work contributes to our understanding of how aging organisms react and adapt to stress and can provide the groundwork for future therapeutic aging interventions.

## 2. Materials and Methods

### 2.1. Fly Stocks and Maintenance

All fly stocks were maintained on solid cornmeal agar (BF Formula, Genesee Scientific) and kept at 25 °C under a 12 h:12 h light–dark cycle. *WRNexo^Δ^* null mutants were created as described in Bolterstein et al. [38]. Matched *w^1118^* flies serve as the genetic wildtype control. Deficiency stocks *Df(3R)BSC509* and *Df(3R)Exel6178* (obtained through Bloomington Drosophila Stock Center) were used in some experiments in *trans* to confirm that observed phenotypes were not due to second site mutations. For all experiments, flies were allowed to mate for 24–48 h following eclosion and then separated by sex under CO_2_ anesthetization. Flies were then transferred directly to an experiment or aged for 14–28 days. Therefore, all experiments contained flies that are 2–3-days old unless otherwise specified. For aging experiments, flies were maintained in vials of approximately 20 individuals and transferred to new food every 2–3 days for the duration of aging. For the sake of continuity, only female data is presented in main body of this manuscript; male data is available in the supplement.

### 2.2. Larval Stress Assays

We used a modified mutagen sensitivity assay [39] to assess relative survival of larvae exposed to either oxidative stress or elevated ambient temperature. Briefly, heterozygous males and females were mated and allowed to lay eggs for 3 days at 25 °C (brood 1). The mated parents were then transferred into a second vial to lay for an additional 2 days (brood 2). To induce oxidative stress in larvae, brood 1 vials were treated with 250 μL 20 mM paraquat or 5% hydrogen peroxide dissolved in water 1 day after the transfer of parents. The brood 2 vials served as the controls and were treated with water only. For elevated ambient temperature experiments, brood 1 vials were moved to 29 °C following the removal of parents, while brood 2 vials remained at 25 °C to serve as temperature controls. Relative survival of larvae to adulthood was calculated as percentage of viable homozygous progeny in treated vials (brood 1) divided by percentage of viable homozygous progeny in control vials (brood 2). Each vial served as an experimental replicate and each experiment was repeated on at least two separate occasions.

### 2.3. Adult Oxidative Stress Assays

Newly eclosed males and females were mated for 24–48 h and then separated by sex under CO_2_ anesthesia before transferring groups of 10 flies to empty polystyrene vials. Flies were maintained at 25 °C for three hours for starvation and to lessen the CO_2_ effect. The flies were then transferred to vials containing ¼ of a 9 cm Whatman #1 filter paper treated with 250 μL 5% sucrose in water and/or 20 mM paraquat and/or 5% hydrogen peroxide. Flies were returned to 25 °C and death was recorded every 6–12 h. Kaplan–Meier lifespan curves were created for each biological replicate (2–3 per genotype/age/treatment). To compare lifespans while minimizing small changes due to environmental differences, median death for each experiment was normalized by dividing the time of death of each individual fly by the median lifespan for that genotype/sex/treatment in that individual experiment. Statistical significance was determined using 2-way ANOVA and Šídák’s multiple comparisons post hoc test.

### 2.4. Antioxidant Activity

Crude protein was extracted from groups of 50 flies separated by sex and genotype as described in Emery et al. [40] and quantified using the Pierce BCA Protein Assay Kit. 100 μg of crude protein extract in 300 μL protein extraction buffer was added in triplicate to a 96-well plate. The stable free radical, DPPH, was dissolved in methanol and added to each well to achieve a final concentration of 250 μM DPPH. Protein extraction buffer served as a blank. Samples were incubated at 37 °C for 30 min and then analyzed at 517 nm wavelength by spectrophotometer to measure reduction in DPPH in a colorimetric assay. Percent DPPH reduced was calculated as mean absorbance of the blank minus mean absorbance of the sample divided by mean absorbance of the blank. Statistical significance was determined using 2-way ANOVA and Šídák’s multiple comparisons post hoc test.

### 2.5. Larval Buoyancy

The larval buoyancy assay [41] was used to determine changes in body fat in vitamin C (ascorbic acid)-treated larvae. Flies were allowed to lay eggs for 24 h in vials containing standard cornmeal agar. On day three, vials were treated with either 250 μL 10 mM ascorbic acid or water. Then, 2–3 days later, third instar wandering larvae were removed from vials, rinsed in PBS, and sets of 20–30 wandering larvae were transferred to vials containing 4 mL of 10% sucrose in PBS. Larvae were agitated and allowed to settle before scoring, floating, defined as larvae at the surface of the liquid. Statistical significance showing the impact of ascorbic acid on the percentage of floating larvae was determined by Fisher’s Exact Test.

### 2.6. Drosophila Activity Monitors (Thermal Stress, Starvation, and Sleep Analysis)

Continuous monitoring of locomotor activity and hourly activity averages were assessed using *Drosophila* Activity Monitors (DAM2, TriKinetics) as described previously [37]. Briefly, flies were allowed to mate for 24–48 h post-eclosion before being moved to DAMs. Single flies were continually monitored in 1 min intervals over a 4-day (starvation and sleep) or 6-day (temperature) period using the TriKinetics software. There were 2–4 independent experiments performed for each genotype and condition tested, each containing 8–32 individual flies.

DAM data was analyzed using a counting macro program as previously described [42]. Sleep bouts are defined as instances of ≥5 min of inactivity [43]. Average bout length was defined as the average length of these periods of inactivity over the course of the experiment, while average bout number was calculated as the number of sleep bouts per day. Sleep values were plotted and 2-way ANOVA followed by Dunnett’s multiple comparisons post hoc tests were performed using GraphPad prism version 9.0 (San Diego, CA, USA).

Starvation lifespan analysis also utilized the DAM activity profiles. Data were aggregated for each individual fly by day and the timing of the last probable activity episode (excluding false positives) was subtracted from the time of DAM monitor activation. False positives were removed from the end of the record by excluding days after the main activity bout with <20 beam breaks/day. All data were processed and analyzed using the R 4.0.2 statistical software [44] (all code and data are available upon request). Overall activity and individual hourly activities were compared using a Kruskal–Wallis test and activity distribution was compared using a Kolmogorov–Smirnov test. Individual lifespans were compared using ANOVA.

## 3. Results

### 3.1. Exogenous Stressors

The response of *WRNexo^Δ^* to stress varied based on age and type of stressor. We chose three exogenous stressors: oxidative stress, elevated ambient temperature, and starvation, which are well supported in the literature as eliciting a stress response in flies [9,10,11,12,13,14,15] and are mechanistically connected to WRN. Oxidative stress, in the form of exposure to hydrogen peroxide (H_2_O_2_) and paraquat, was chosen because of WRN’s involvement in responding to oxidative stress [45]. Elevated ambient temperature has been shown to cause physiological and behavioral changes in *Drosophila* [46,47], increase mutation frequency [15], and increase ROS levels [48], providing different mechanisms of DNA damage and stress response. We also tested starvation as a stressor as it has been shown to not only deprive organisms of nutrients, but also reduce DNA repair enzyme functionality due to lower ATP production [49] and to further examine low body fat in *WRNexo^Δ^* [37].

#### 3.1.1. Oxidative Stress

**Figure 1:**
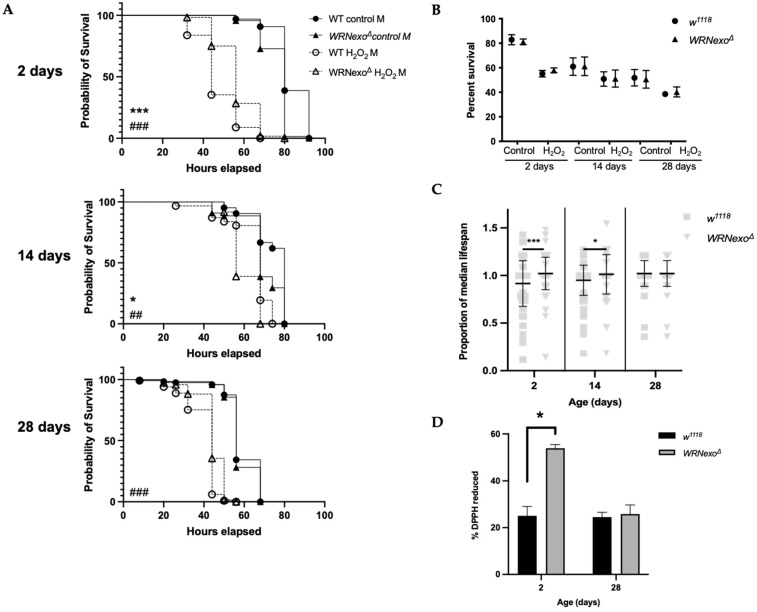
*WRNexo^Δ^* females are not sensitive to exogenous oxidative stress regardless of age. (**A**) Representative Kaplan–Meyer survival curves for adult survival following hydrogen peroxide exposure. (Mantel–Cox log-rank between *w^1118^* and *WRNexo^Δ^* controls: * *p* < 0.05, *** *p* < 0.001; Mantel–Cox log-rank between H_2_O_2_-treated *w^1118^* and *WRNexo^Δ^*: ^##^ *p* < 0.01, ^###^ *p* < 0.001, *n* = 38–130). (**B**) Summary data depicting mean lifespan of flies exposed to H_2_O_2_. (2-way ANOVA, *n* = 2–3 independent experimental replicates/per condition ± SEM). (**C**) Normalized lifespans following 5% hydrogen peroxide showed resistance of younger, but not aged *WRNexo^Δ^* females (2-way ANOVA, * *p* < 0.05, *** *p* < 0.001, ±SD, *n* = 128–259 females from 2 to 3 independent experimental replicates). (**D**) Crude protein extracts from young *WRNexo^Δ^* adults had the greatest neutralization effect on the stable free radical DPPH demonstrating higher antioxidant activity (paired *t*-test, * *p* < 0.05, ±SEM).

We first tested larval response to the oxidative stress reagents H_2_O_2_ and paraquat and found that *WRNexo^Δ^* larvae were not sensitive to exogenous oxidative stress relative to their heterozygous controls (Figure S6). We then investigated adult sensitivity to oxidative stress at various ages by exposing *w^1118^* and *WRNexo^Δ^* females to H_2_O_2_ and paraquat at 2-, 14-, and 28-days old. As expected, H_2_O_2_ reduced lifespan in both *w^1118^* and *WRNexo^Δ^* for all ages tested (Figure 1A,B and Figures S1–S3; Table S1). Though not apparent in the summarized lifespan data (Figure 1B), individual lifespan experiments showed that while *WRNexo^Δ^* females had a shorter lifespan than *w*^111^ controls, H_2_O_2_-treated *WRNexo^Δ^* females showed a longer lifespan compared to identically-treated *w^1118^* (Figure 1A and Figures S1–S3), suggesting that *WRNexo^Δ^* females may be slightly resistant to stress induced by H_2_O_2_. We then normalized data to account for environmental differences between experiments and again saw H_2_O_2_ resistance in 2-day and 14-day old *WRNexo^Δ^* females that diminished by day 28 (Figure 1C), demonstrating that age is a factor in stress resistance. Young paraquat-treated *WRNexo^Δ^* showed increased lifespan compared to untreated controls; however, the difference between paraquat-treated *WRNexo^Δ^* and *w^1118^* lifespan was not significant (Figure S5).

Because a high antioxidant environment can protect against oxidative stress, we tested intrinsic antioxidant capacity in young and old *WRNexo^Δ^*. Using the stable free radical DPPH, we found that young *WRNexo^Δ^* females had higher antioxidant activity compared to age-matched *w^1118^* controls; however, old (28 day) *WRNexo^Δ^* females had similar antioxidant activity to *w^1118^* (Figure 1D). Male flies showed similar results (Figure S4). Therefore, while it is likely that increased intrinsic antioxidant capacity helped to protect *WRNexo^Δ^* as young adults, this protection may not have been extended to older flies.

#### 3.1.2. Response to Non-Optimal Ambient Temperature

**Figure 2:**
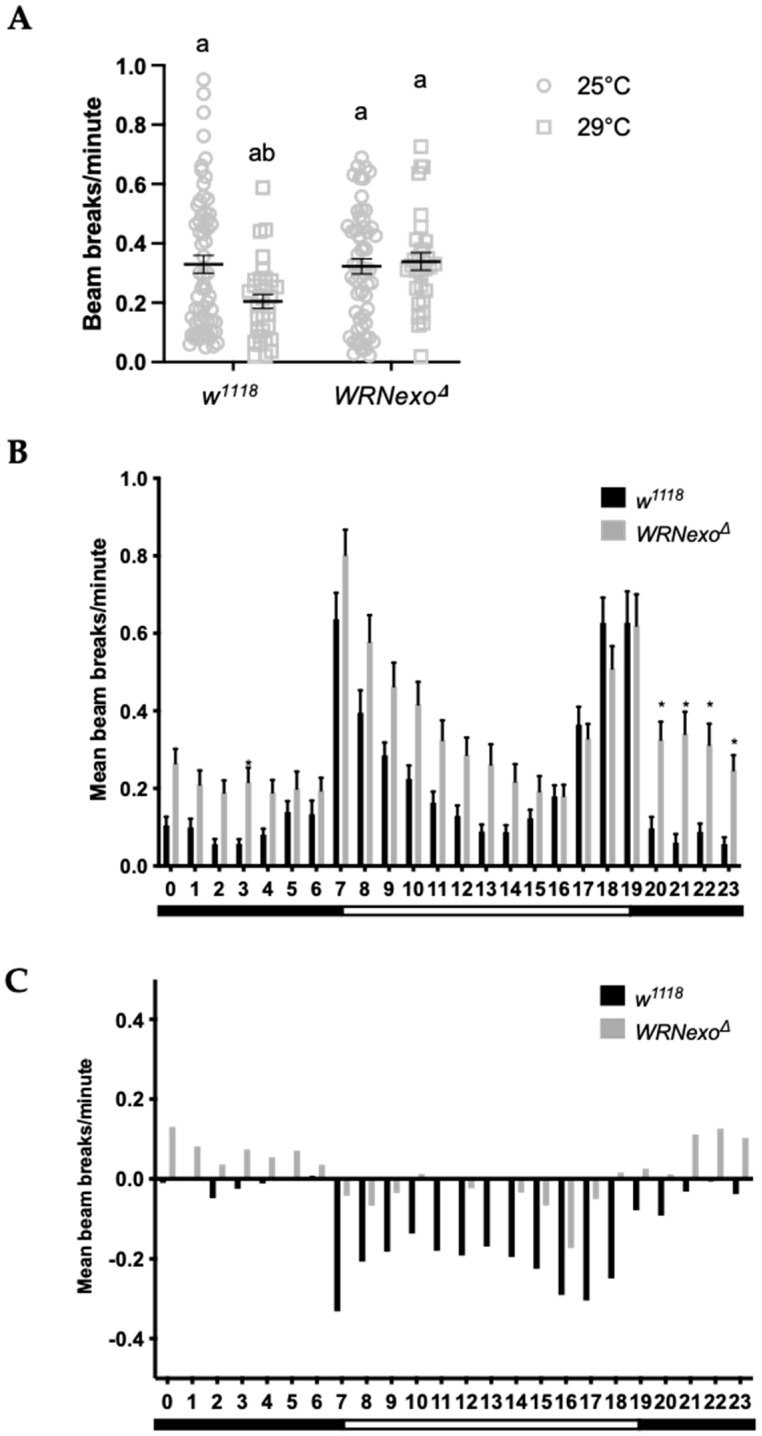
Activity in young *WRNexo^Δ^* females increases at elevated ambient temperature. (**A**) While overall activity declines for *w^1118^* females at 29 °C, *WRNexo^Δ^* activity does not change (Kruskal–Wallis test, letters denote statistically significant groups, *p* < 0.0001, ±SEM). (**B**) Activity peaks are evident at light transition periods represented by the black and white bars. There was a significant difference in activity distribution between *w^1118^* and *WRNexo^Δ^* at 29 °C (Kolmogorov–Smirnov test, *p* < 0.0001) and significant differences between mean hourly intervals from hour 20–23 (Kruskal–Wallis test, * *p* < 0.01, ±SEM, 25 °C: *w^1118^ n* = 62, *WRNexo^Δ^* *n* = 62; 29 °C: *w^1118^ n* = 32, *WRNexo^Δ^* *n* = 31. (**C**) Change in hourly activity levels for each genotype was calculated by subtracting activity levels at 25 °C from activity levels at 29 °C. Compared to *w^1118^*, *WRNexo^Δ^* females showed an increase in activity at night.

*WRNexo^Δ^* larvae raised at a low (18 °C) or elevated (29 °C) ambient temperature showed similar relative survival to adulthood as controls (Figure S7), demonstrating that like oxidative stress, non-optimal ambient temperature did not impact larval survival. We then measured young adult activity at elevated ambient temperature and found that as expected [50], total activity decreased in young *w^1118^* females, mostly through decreased daytime activity (Figure 2A,B). However, rather than showing a temperature-induced decrease in activity, young *WRNexo^Δ^* females showed similar levels of activity at both 25 °C and 29 °C (Figure 2A). Activity differences were not observed in males (Figure S8). Activity at an elevated temperature was further examined through the hourly average activity profile, which confirmed greater overall activity of *WRNexo^Δ^* females compared to *w^1118^* and also demonstrated that *WRNexo^Δ^* had unusually high nighttime activity (Figure 2B,C). Together, these data indicate decreased rest in *WRNexo^Δ^* flies exposed to elevated ambient temperature which suggests stress sensitivity in adults.

#### 3.1.3. Starvation

**Figure 3:**
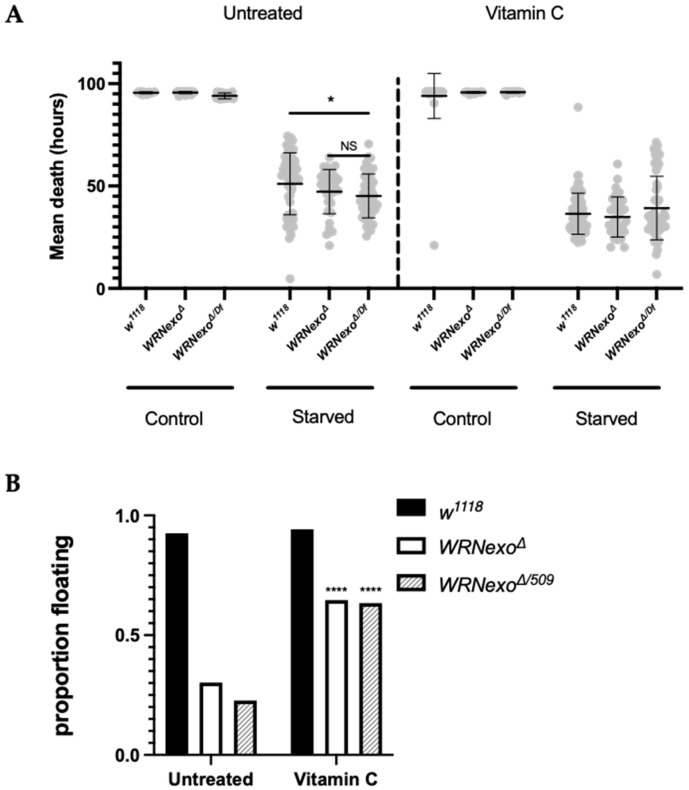
*WRNexo^Δ^* show sensitivity to starvation that is impacted by treatment with vitamin C. (**A**) *WRNexo* females have shorter mean lifespans under starvation compared to age-matched *w^1118^* controls. Vitamin C-treated flies have similar lifespans regardless of genotype (2-way ANOVA and Tukey’s multiple comparisons post hoc test, * *p* < 0.05, ±SD, *n* = 16–80 flies/genotype). Df = deficiency strain: *WRNexo^Δ/Df509^* for untreated experiments and *WRNexo^Δ/Df6178^* for experiments with vitamin C treatment. (**B**) Vitamin C treatment leads to higher levels of body fat in *WRNexo^Δ^* larvae (Fisher’s exact test, **** *p* < 0.0001, Untreated: *w^1118^ n* = 660, *WRNexo^Δ^* *n* = 288; *WRNexo^Δ/Df509^* *n* = 207; Vitamin C-treated: *w^1118^ n* = 275, *WRNexo^Δ^* *n* = 325; *WRNexo^Δ/Df509^* *n* = 71, where n is total larvae).

We hypothesized that *WRNexo^Δ^* would be more sensitive to starvation stress based on our previous work showing that *WRNexo^Δ^* larvae have lower body fat and *WRNexo^Δ^* adult females have lower body weight in comparison to *w^1118^* controls [37]. Indeed, we found that both male and female *WRNexo^Δ^* adults had shorter lifespans under starvation compared to *w^1118^* controls (Figure 3A and Figure S8). To further explore if starvation sensitivity was related to body fat and weight, we increased larval body fat of *WRNexo^Δ^* by treating second instar larvae with 10 mM ascorbic acid (vitamin C) (Figure 3B). Vitamin C treatment also increased larval and adult dry mass approximately twofold from previously reported untreated values [37] in a proportional manner in which *WRNexo^Δ^* adults were smaller than *w^1118^* (Table 1). Vitamin C treatment resulted in no difference in lifespan between starved *w^1118^* and *WRNexo^Δ^* females, which suggests that higher body fat rescued starvation sensitivity in *WRNexo^Δ^* compared to *w^1118^* (Figure 3B). However, an alternate explanation may lie in our observed vitamin C toxicity: Vitamin C treatment resulted in a shorter lifespan for all genotypes, possibly owing to its pro-oxidant qualities at high doses [51,52]. However, *WRNexo^Δ^* flies were less affected by vitamin C toxicity as shown by a smaller difference in lifespan between untreated and vitamin C-treated flies. Vitamin C reduced lifespan by 14.6 h (−28.7% change) in *w^1118^* females compared to a 12.4 h reduction in lifespan (−26.2% change) in *WRNexo^Δ^*. While vitamin C also reduced lifespan in male flies, starvation sensitivity was unaffected (Figure S9).

### 3.2. Sleep Analysis

**Figure 4:**
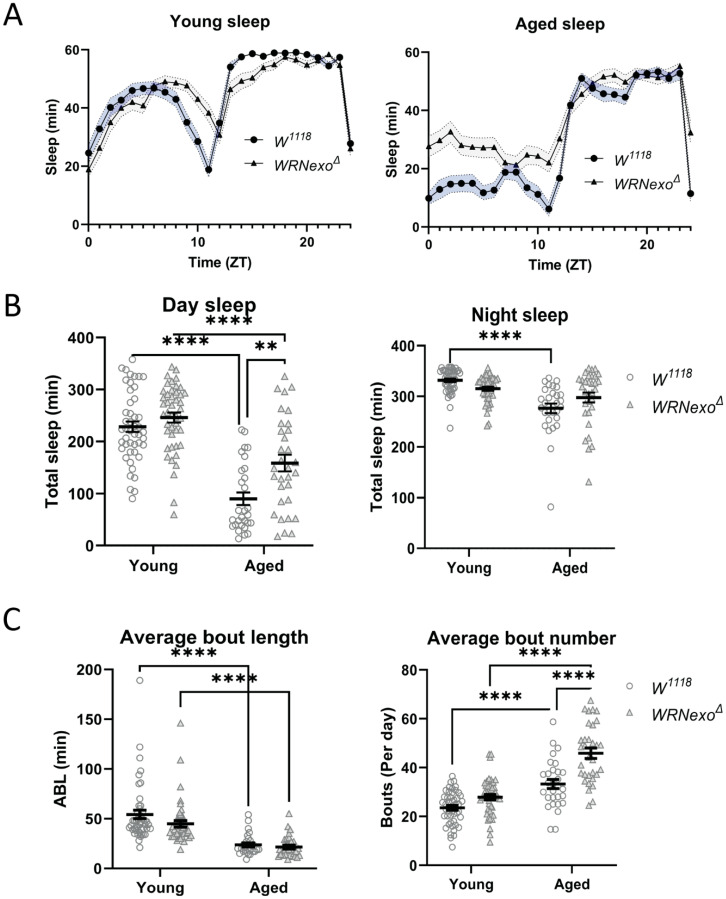
*WRNexo^Δ^* display age-dependent differences in total sleep and composition. (**A**) Young *w^1118^* and *WRNexo^Δ^* flies exhibit similar levels of sleep throughout the day, while aged *WRNexo^Δ^* flies exhibit greater sleep during the light phase compared to aged *w^1118^* controls. (**B**) Both aged *w^1118^* and *WRNexo^Δ^* flies exhibit decreased sleep during the day compared to young flies and aged *WRNexo^Δ^* flies display greater sleep than age-matched *w^1118^*. (**C**) Aged *WRNexo^Δ^* show greater sleep fragmentation compared to *w^1118^* flies. While *WRNexo^Δ^* and *w^1118^* exhibit similar average bout length in both young and aged flies, aged *WRNexo^Δ^* exhibit significantly greater bouts per day compared to age matched *w^1118^* controls. (2-way ANOVA, ** *p* < 0.01, **** *p* < 0.0001, ±SD).

Organisms often respond to stress through physiological and behavioral changes [53]. We had previously reported that aged *WRNexo^Δ^* flies showed lower activity compared to age-matched *w^1118^* controls, a phenotype likely related to higher muscular degeneration [37]. We expanded upon this behavioral analysis by investigating changes in sleep, another behavior that degrades in response to both age and chronic cellular damage [54,55,56]. We hypothesized that, because of their accelerated aging and increased levels of cellular damage [37], *WRNexo^Δ^* would exhibit increased age-related deterioration of sleep. *w^1118^* female flies exhibited decreased sleep that was more fragmented with age (Figure 4A–C), which is consistent with published reports [56,57]. Aged *WRNexo^Δ^* flies exhibited a similar decrease in sleep accompanied by shorter, more numerous bouts (Figure 4A–C) indicating that age-related sleep changes also occurred in this mutant. There was little difference in total sleep between young *WRNexo^Δ^* and *w^1118^* controls (Figure 4B) with only a minimal increase in bout number suggesting increased fragmentation (Figure 4C). Surprisingly, aged *WRNexo^Δ^* flies displayed an increase in sleep primarily in the daytime compared to *w^1118^* (Figure 4B). This difference was driven primarily by a near 50% increase in bout number (aged *w^1118^*—33 bouts/day, aged *WRNexo^Δ^*—46 bouts per day). Conversely, aged *WRNexo^Δ^* males showed decreased sleep compared to age-matched *w^1118^* controls (Figure S10), demonstrating sex-dependent differences in sleep behavior.

## 4. Discussion

It is well accepted that organisms’ ability to adapt to stress declines with age [7] and that stress response is just one aspect of aging out of many [2]. What is less understood is how the various mechanisms of aging interact with each other. Here, we show phenotypic data demonstrating a multifaceted intersection between stress adaptation and metabolism in a progeroid fly model. The stress response exhibited by *WRNexo^Δ^* mutants differed by age and type of stressor: adult *WRNexo^Δ^* were resistant to exogenous oxidative stress when young, but lost their resistance to oxidative stress as they aged possibly due to changes in antioxidant capacity. Conversely, activity of *WRNexo^Δ^* adults was altered in elevated ambient temperature suggesting stress sensitivity. *WRNexo^Δ^*’s sensitivity to starvation could be ameliorated through vitamin C treatment, which may further link WRN deficiency to elevated levels of endogenous oxidative stress as well as metabolic dysfunction. Because *WRNexo* in flies demonstrate similar molecular and preventative aging functions as human WRN [33,34,36,37], our data suggest an exonuclease-specific role for WRN in responding to stress that has not previously been defined. Further, the correlation of age-dependent protective mechanisms of higher antioxidant activity in young *WRNexo* mutants and higher levels of sleep in old mutants suggests adaptive responses to stress, possibly through the modulation of oxidative stress.

WRN is required for proper cellular redox potential, which is essential for maintaining the low physiological levels of ROS needed for cellular signaling of stress responses and metabolic regulation [58,59]. WS cells and animal models have shown increased ROS [60,61], and/or decreased expression/activity of antioxidant proteins [61,62], which may limit ROS signaling abilities. Consistent with our observed high antioxidant activity in *WRNexo^Δ^*, elevated levels of small molecule antioxidants, specifically ascorbic acid (vitamin C) and uric acid, have been observed in WS patients [63], suggesting the need for antioxidant defenses to maintain redox homeostasis. To that effect, continuous feeding of vitamin C both decreased ROS levels as well as rescued age-related pathologies in mice deficient in WRN helicase, including increased fat storage in adipose tissues, restored genomic integrity, and reduced inflammation while showing no effect on wild-type mice [60]. Similarly, vitamin C treatment has also been shown to extend lifespan and alter transcription of metabolic genes in WRN helicase-deficient *C. elegans* (*wrn-1*) [64]. Our data are exonuclease-specific and show that vitamin C increases larval body fat in *WRNexo* mutants, but decreases mutants’ sensitivity to starvation stress. It is possible that vitamin C reduces levels of ROS in *WRNexo* mutants back to the physiological levels needed to restore proper metabolic regulatory signaling. Conversely, higher vitamin C toxicity in *w^1118^* may indicate a redox imbalance where ROS levels are too low to maintain stress response homeostasis.

Oxidative stress resistance and high activity in elevated ambient temperature, as shown here in *WRNexo^Δ^* adults, may also indicate higher levels of cellular damage; oxidative stress-induced mutations may allow cells to bypass anti-proliferation signaling, potentially leading to tumorigenesis and cancer [65]. This concept is shown in WS patients who display significant genomic instability and an elevated cancer risk [22]. WS fibroblasts are resistant to oxidative stress and show continued proliferation and senescence avoidance [66] and an absence of gene expression changes (cell cycle and proliferation, lipid metabolism, nucleic acid metabolism, and vesicle and protein transport) when treated with hydrogen peroxide [61]. Together these studies indicate that high levels of stress/damage intrinsically present in WRN-deficient cells may keep DNA damage responses acting at full capacity. Therefore, additional DNA damage caused by exogenous oxidative stress or elevated ambient temperature, cannot be repaired, leading to a persistent cancer cell phenotype [22]. While we did not measure oxidative stress-induced DNA damage in this study, it is possible that the slight increase in lifespan under exogenous oxidative stress and abnormal activity in elevated temperature is due to damaged cell cycle regulation, allowing flies to live despite cellular damage.

Tumor cells are also marked by metabolic changes that may drive cancer progression [67]. In comparison with normal tissue, cancer cells have been shown to have high cellular metabolism leading to an increase in ROS and subsequent increased macromolecular damage [67]. The increased tumor risk inherent to *WRNexo^Δ^* [37] may suggest similarities with a tumor microenvironment, thereby promoting increased metabolism. It is therefore possible that low larval body fat and adult starvation sensitivity in *WRNexo^Δ^* may be due to higher basal metabolism and subsequent lower reserves of available energy. To this point, stressed flies show reduced energy stores [68,69] possibly due to higher basal metabolism in responding to cellular damage [15] and/or a stress-induced switch to non-ATP producing metabolic mechanisms [68]. Therefore, low energy stores may cause higher activity in *WRNexo^Δ^* due to more time spent foraging for food [68]. However, activity levels of both young and old *WRNexo^Δ^* raised in normal temperature conditions did not show an increase in activity, suggesting that potential metabolic changes are stress-induced.

In addition to maintaining normal activity levels, aged *WRNexo^Δ^* adults sleep more than wild-type controls, especially during the day, although their sleep remains highly fragmented. While increased sleep fragmentation in older flies is the general consensus, overall sleep levels have been shown to either decline with age [57], or similar to *WRNexo^Δ^*, increase [56,70]. Further, changes in sleep are correlated with infection [71], oxidative stress [56,72,73,74], and low nutrient availability [75] suggesting that sleep may provide a protective mechanism against stress. Sleep has been shown to clear harmful metabolites and ROS from glial and hemolymph cells thereby preventing cellular damage and disease such as Alzheimer’s [74,76,77,78]. Additionally, because the metabolic demands are less on the brain during sleep, sleep may be key in replenishing neuronal energy stores [74,79] thereby preventing age-related cognitive decline. Therefore, we suggest that *WRNexo^Δ^* may not only sleep more during the day to make up for poor quality sleep (high fragmentation), but also that increased sleep in these flies may serve as a protective mechanism to respond to DNA and cellular damage that accumulate during aging.

Our observations that vitamin C rescues low body fat in *WRNexo^Δ^* may further strengthen the link between sleep, altered locomotor activity, and metabolism as it is possible that low body fat may indicate abnormal fat body physiology. The fly fat body has been linked to various physiological processes, such as egg laying and detoxification [80], and most germane to this study, altered metabolic gene expression and sleep regulation [81,82]. Because changes in the larval fat body may impact adult physiology [83,84], low larval body fat may cause permanent changes in cellular metabolism and sleep regulation that manifest as altered sleep and activity patterns in mutant adults.

WRN’s role in regulating cellular metabolism may also be directly linked to preventing mitochondrial dysfunction, which is one of the hallmarks of aging [1]. WRN depletion has been shown to increase cellular levels of the hypoxia regulatory protein HIF-1, which in turn increase levels of mitochondrial ROS [85]. Additionally, WS and WRN-deficient cells show increased expression of metabolic genes that protect against oxidative stress [59,61,86,87]. Fatty acid accumulation may result in increased ROS as beta oxidation is more energy intensive and therefore releases a greater number of mitochondrial ROS [88]. Mitochondrial dysfunction has been directly implicated as a cause of WS symptoms in a study showing that WS models are depleted in the essential metabolic reducing molecule NAD+ [86]. WS and WRN-deficient human cells showed higher mitochondrial ROS levels, lower mitochondrial membrane potential, decreased mitochondrial content, and decreased cellular ATP levels. NAD+ augmentation rescues mitochondrial dysfunction phenotypes in WS cell models and extends lifespan in *wrn-1(gk99) C. elegans* and WRN KD *Drosophila* (*WRNexo^RNAi^*) [86]. While it is likely that our *WRNexo^Δ^* flies are also experiencing mitochondrial dysfunction, additional work is required to directly make this link.

We postulate that the stress responses observed in our *WRNexo* mutants cannot be attributed to any one mechanism of aging, but instead indicate a systemic physiological response resulting in stress protection in young animals. Possible stress response mechanisms in *WRNexo* mutants may include disruption of cellular redox potential and ROS metabolic regulatory signaling homeostasis, increased DNA damage causing a tumor-like cellular persistence, and using sleep to repair and protect against age-related cellular damage. Together, it is possible that *WRNexo* mutants have elevated intrinsic oxidative stress, which may stimulate other protective mechanisms leading to a beneficial hormetic effect. The benefits of mild stress on lifespan and stress resistance has been shown in numerous aging models in a manner dependent on frequency and duration of exposure, as well as the age in which the stressor was applied [16,89,90,91,92]. While mild cold stress extends lifespan for flies up to 3 weeks of age, the benefits diminish in older flies [16]. Therefore, intrinsic stress in older *WRNexo* mutants may not elicit a strong enough protective stress response to increase antioxidant activity at that time and instead may increase sleep to protect against damage. The mitochondrial free radical theory of aging posits that the accumulation of ROS during aging drives aging phenotypes and disease [93]. However, more recent research suggests that an increase in ROS is a symptom of aging and not a cause [94]. In fact, in light of redox imbalance in WRN-deficient models [60,61,62], and WRN’s role in preventing mitochondrial dysfunction [86], this effect may indicate that WRN may be involved in maintaining mitohormesis, in which elevated mitochondrial ROS stimulate antioxidant expression and other protective mechanisms [20]. Future studies to investigate mitohormesis in WS models will be bolstered by improved methods in determining the level and cellular location of ROS as that can greatly impact the hormetic effect [95].

In conclusion, *WRNexo* in flies may be involved in protecting against high levels of endogenous stress due to its roles in maintaining genomic stability and proper cellular redox potential. In the absence of *WRNexo*, the stress incurred elicits a systemic response that may lead to greater stress tolerance. Furthermore, because *WRNexo* contains only the exonuclease domain of WRN, the observed hormetic responses to stress, and aging phenotypes in *WRNexo^Δ^*, indicate an exonuclease-dependent role for WRN in responding to stress. This work further validates *WRNexo^Δ^* flies as a model for studying mechanisms of aging and progeroid disease. Future work using this model can uncover potential therapeutic and preventative approaches that can contribute to treating human disease.

## Figures and Tables

**Table 1:** Vitamin C-treated larval and adult dry mass/10 individuals (mg).

Treatment	Genotype	Larvae	Adult Male	Adult Female
Untreated [37]	*w^1118^*	4.2 ± 0.5	1.7 ± 0.3	2.6 ± 0.4
	*WRNexo^Δ^*	3.5 ± 0.8	1.5 ± 0.3	2.0 ± 0.5 *
Vitamin C	*w^1118^*	8.2 ± 1	4.0 ± 0.3	5.0 ± 0.5
	*WRNexo^Δ^*	7.5 ± 0.8	2.0 ± 0.3 ****	3.9 ± 0.5 ****

*n* = 10 groups of 10 individuals per sex/genotype; * *p*< 0.05, **** *p* < 0.0001 compared to same sex *w^1118^* control within treatment group by Student’s *t*-test, ±SD.

## Data Availability

The data presented in this study are available on request from the corresponding author.

## Supplementary Materials

The following are available online at https://www.mdpi.com/article/10.3390/biom11121868/s1, Figure S1: Kaplan–Meyer survival curves for 2-day-old adult hydrogen peroxide sensitivity experiments including *WRNexo^Δ^* and *w^1118^* flies, Figure S2: Kaplan–Meyer survival curves for 14-day-old adult female hydrogen peroxide sensitivity experiments including *WRNexo^Δ^* and *w^1118^* flies, Figure S3: Kaplan–Meyer survival curves for 28-day-old hydrogen peroxide sensitivity experiments including *WRNexo^Δ^* and *w^1118^* flies, Figure S4: Adult male *WRNexo^Δ^* are not sensitive to hydrogen peroxide at 2 and 28 days, but do show high antioxidant activity in young flies, Figure S5: Kaplan–Meyer survival curves for young adult *WRNexo^Δ^* in response to paraquat, Figure S6: Oxidative stress does not impact survival of *WRNexo^Δ^* larvae, Figure S7: Non-optimal ambient temperature does not impact survival of *WRNexo^Δ^* larvae, Figure S8: Elevated ambient temperature does not impact overall activity of *WRNexo^Δ^* males, Figure S9: *WRNexo^Δ^* males show similar starvation sensitivity as *WRNexo^Δ^* females that is not impacted by vitamin C treatment, Figure S10: Sleep profile in *WRNexo^Δ^* males.
