# Supplementary material for: Characterization of Stress Responses in a Drosophila Model of Werner Syndrome
Source: Biomolecules. 2021 Dec 12;11(12):1868. doi: 10.3390/biom11121868 (PMC8699552; doi:10.3390/biom11121868)
Supplement: Supplementary file 1 [file biomolecules-11-01868-s001.zip › biomolecules-1463595-supplementary.pdf]

Supplementary Materials

Table S1: Raw lifespan data for adult oxidative stress assays

Age	Treatment	Genotype	Sex	Trial #	Date	n	me-dian	mean
young	Untreated	w1118	M	1	10/23/16	38	80	78.4
young	Untreated	WRNexo^delta	M	1	10/23/16	59	80	74.1
young	H2O2 5%	w1118	M	1	10/23/16	50	56	52.4
young	H2O2 5%	WRNexo^delta	M	1	10/23/16	69	56	55.3
young	Untreated	w1118	F	1	10/23/16	38	80	76.2
young	Untreated	WRNexo^delta	F	1	10/23/16	57	80	82.9
young	H2O2 5%	w1118	F	1	10/23/16	50	56	57.2
young	H2O2 5%	WRNexo^delta	F	1	10/23/16	70	56	61.8
young	Untreated	w1118	M	2	11/7/16	89	80	76.0
young	Untreated	WRNexo^delta	M	2	11/7/16	70	68	66.8
young	H2O2 5%	w1118	M	2	11/7/16	97	44	46.6
young	H2O2 5%	WRNexo^delta	M	2	11/7/16	59	44	48.5
young	Untreated	w1118	F	2	11/7/16	98	80	82.2
young	Untreated	WRNexo^delta	F	2	11/7/16	70	80	76.5
young	H2O2 5%	w1118	F	2	11/7/16	96	56	49.9
young	H2O2 5%	WRNexo^delta	F	2	11/7/16	60	56	56.6
young	Untreated	WRNexo^delta	M	3	5/15/17	99	80	72.5
young	H2O2 5%	WRNexo^delta	M	3	5/15/17	106	56	49.8
young	Untreated	WRNexo^delta	F	3	5/15/17	130	80	76.0
young	H2O2 5%	WRNexo^delta	F	3	5/15/17	130	56	51.8
young	Untreated	w1118	M	3	5/15/17	41	80	77.6
young	H2O2 5%	w1118	M	3	5/15/17	30	68	65.0
young	Untreated	w1118	F	3	5/15/17	51	92	90.3
young	H2O2 5%	w1118	F	3	5/15/17	50	68	58.4
2 weeks	Untreated	WRNexo^delta	F	1	12/2/16	89	56	58.6
2 weeks	H2O2 5%	WRNexo^delta	F	1	12/2/16	86	44	43.3
2 weeks	Untreated	w1118	F	1	12/2/16	108	68	63.0
2 weeks	H2O2 5%	w1118	F	1	12/2/16	110	56	54.5
2 weeks	Untreated	WRNexo^delta	F	2	5/17/17	40	68	72.1
2 weeks	H2O2 5%	WRNexo^delta	F	2	5/17/17	40	56	58.6
2 weeks	Untreated	w1118	F	2	5/17/17	20	80	75.5
2 weeks	H2O2 5%	w1118	F	2	5/17/17	30	68	65.0
2 weeks	Untreated	WRNexo^delta	F	3	12/11/16	80	50	49.6
2 weeks	H2O2 5%	WRNexo^delta	F	3	12/11/16	79	44	45.1
2 weeks	Untreated	w1118	F	3	12/11/16	50	44	47.7
2 weeks	H2O2 5%	w1118	F	3	12/11/16	50	44	39.4

2 weeks	Untreated	WRNexo^delta	F	2	5/17/17	40	68	72.1
2 weeks	H2O2 5%	WRNexo^delta	F	2	5/17/17	40	56	58.6
2 weeks	Untreated	w1118	F	2	5/17/17	20	80	75.5
2 weeks	H2O2 5%	w1118	F	2	5/17/17	30	68	65.0
4 weeks	Untreated	WRNexo^delta	M	1	12/6/16	110	50	50.0
4 weeks	H2O2 5%	WRNexo^delta	M	1	12/6/16	109	32	28.3
4 weeks	Untreated	WRNexo^delta	F	1	12/6/16	117	56	57.8
4 weeks	H2O2 5%	WRNexo^delta	F	1	12/6/16	118	44	44.4
4 weeks	Untreated	w1118	M	1	12/6/16	138	50	51.0
4 weeks	H2O2 5%	w1118	M	1	12/6/16	131	32	33.2
4 weeks	Untreated	w1118	F	1	12/6/16	119	56	58.5
4 weeks	H2O2 5%	w1118	F	1	12/6/16	117	44	40.3
4 weeks	Untreated	WRNexo^delta	M	2	12/11/16	50	44	41.8
4 weeks	H2O2 5%	WRNexo^delta	M	2	12/11/16	50	26	24.1
4 weeks	Untreated	WRNexo^delta	F	2	12/11/16	40	44	43.4
4 weeks	H2O2 5%	WRNexo^delta	F	2	12/11/16	50	32	36.2
4 weeks	Untreated	w1118	M	2	12/11/16	77	44	45.1
4 weeks	H2O2 5%	w1118	M	2	12/11/16	80	32	29.9
4 weeks	Untreated	w1118	F	2	12/11/16	40	44	45.1
4 weeks	H2O2 5%	w1118	F	2	12/11/16	30	44	36.8
young	Untreated	WRNexo^delta	M	1	11/7/16	70	68	66.8
young	20 mM PQ	WRNexo^delta	M	1	11/7/16	60	44	41.0
young	Untreated	WRNexo^delta	F	1	11/7/16	70	80	76.5
young	20 mM PQ	WRNexo^delta	F	1	11/7/16	70	44	42.5
young	Untreated	w1118	M	1	11/7/16	89	80	76.0
young	20 mM PQ	w1118	M	1	11/7/16	100	32	37.4
young	Untreated	w1118	F	1	11/7/16	98	80	82.2
young	20 mM PQ	w1118	F	1	11/7/16	100	44	41.4
young	Untreated	WRNexo^delta	M	2	11/1/16	60	80	83.4
young	20 mM PQ	WRNexo^delta	M	2	11/1/16	55	56	61.3
young	Untreated	WRNexo^delta	F	2	11/1/16	58	96	90.6
young	20 mM PQ	WRNexo^delta	F	2	11/1/16	68	56	56.8
young	Untreated	w1118	M	2	11/1/16	60	96	95.6
young	20 mM PQ	w1118	M	2	11/1/16	59	56	59.1
young	Untreated	w1118	F	2	11/1/16	50	96	93.1
young	20 mM PQ	w1118	F	2	11/1/16	59	56	56.4

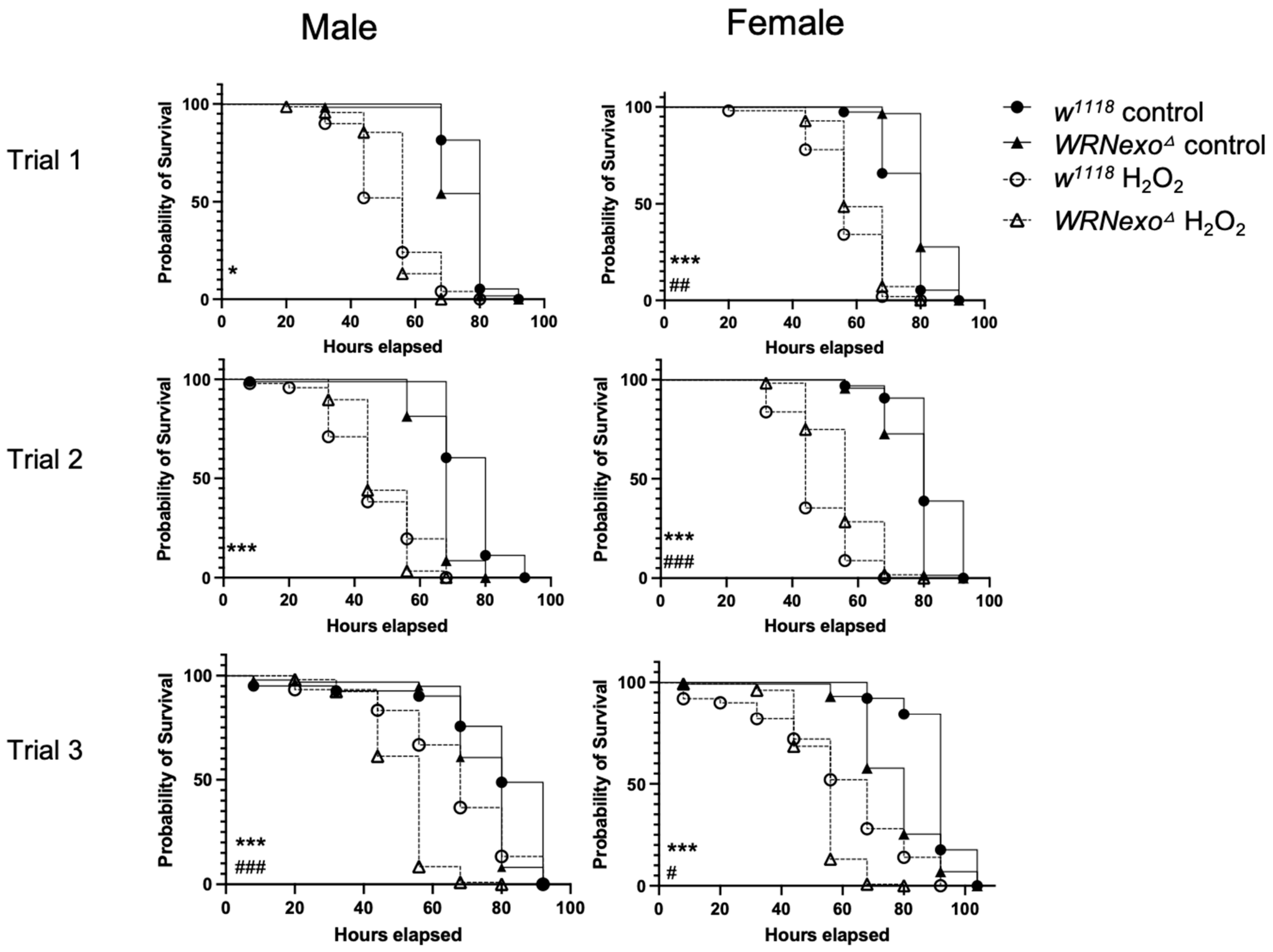

Figure S1: Kaplan-Meyer survival curves for 2 day-old adult hydrogen peroxide sensitivity experiments including *WRNexo*$^{\Delta}$ and *w*^{1118} flies. Mortality was recorded every 6-12 hours. Trial 1: n= 38-70 females and 38-69 males, Trial 2: n= 60-98 females and 59-97 males, Trial 3: n= 50-130 females and 30-100 males (Mantel-Cox log-rank between *w*^{1118} and *WRNexo*$^{\Delta}$ controls: *$p<0.05$, ***$p<0.001$; Mantel-Cox log-rank between H_2O_2-treated *w*^{1118} and *WRNexo*$^{\Delta}$: $^{\#}p<0.05$, $^{\#\#}p<0.01$, $^{\#\#\#}p<0.001$).

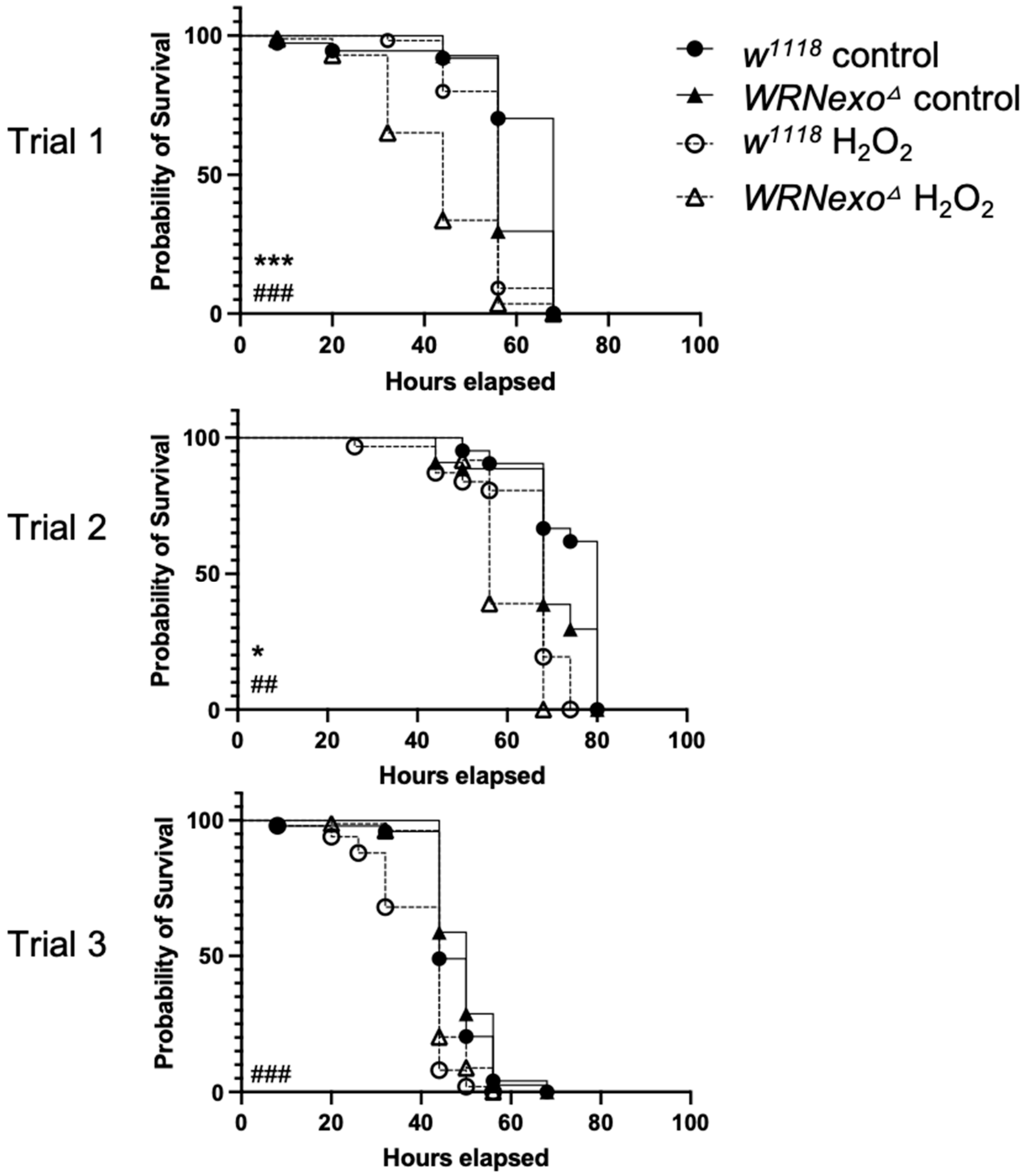

Figure S2: Kaplan-Meyer survival curves for 14-day old adult female hydrogen peroxide sensitivity experiments including *WRNexo*$^{\Delta}$ and *w*^{1118} flies. Mortality was recorded every 6-12 hours Trial 1: n= 84-111, Trial 2: n= 21-44, Trial 3: n= 30-50 (Mantel-Cox log-rank between *w*^{1118} and *WRNexo*$^{\Delta}$ controls: **p*<0.05, ****p*<0.001; Mantel-Cox log-rank between H_2O_2-treated *w*^{1118} and *WRNexo*$^{\Delta}$: ##*p*<0.01, ###*p*<0.001). Male sensitivity to H_2O_2 was not measured.

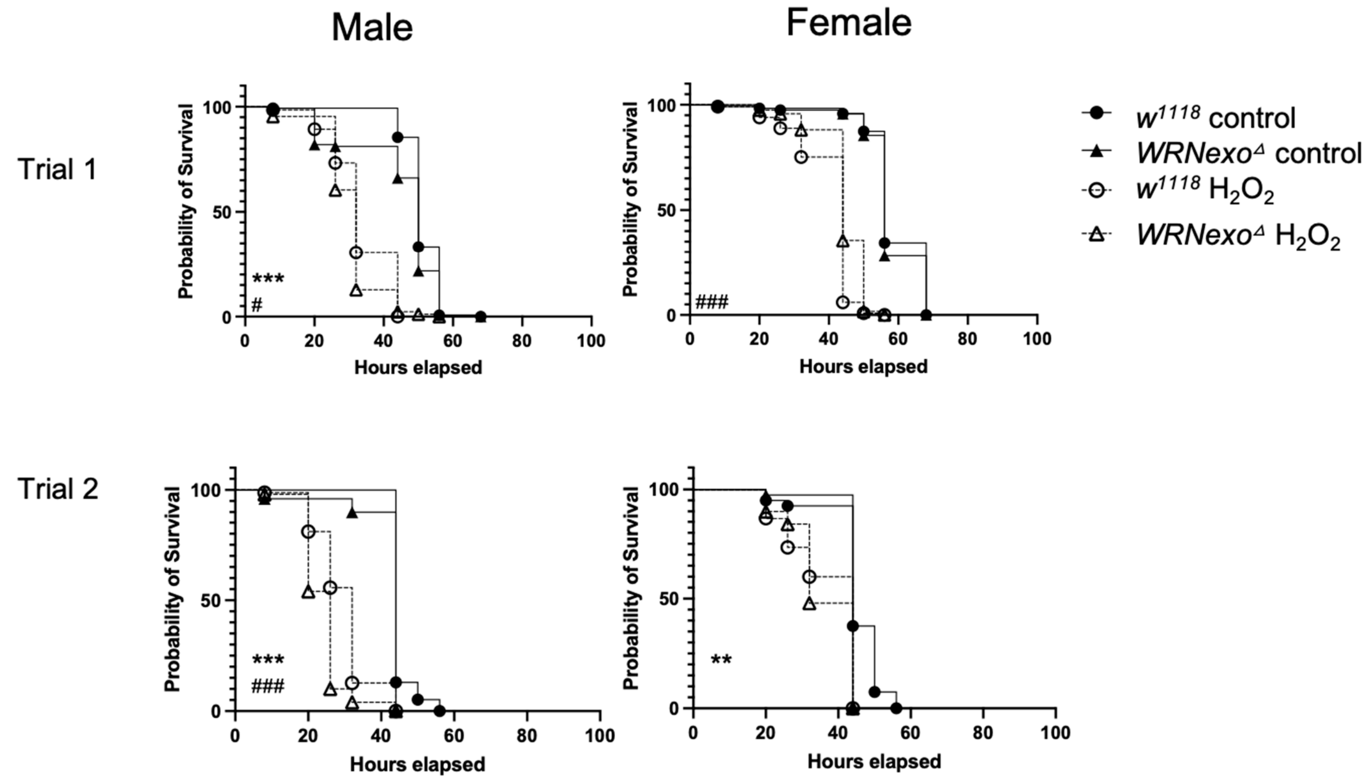

Figure S3: Kaplan-Meyer survival curves for 28 day-old hydrogen peroxide sensitivity experiments including *WRNexo*$^{\Delta}$ and *w*^{1118} flies. Mortality was recorded every 6-12 hours. Trial 1: n= 111-119 females and 86-138 males, Trial 2: n= 30-50 females and 50-79 males (Mantel-Cox log-rank between *w*^{1118} and *WRNexo*$^{\Delta}$ controls: $^{**}p<0.01$, $^{***}p<0.001$; Mantel-Cox log-rank between H_2O_2-treated *w*^{1118} and *WRNexo*$^{\Delta}$: $^{\#}p<0.05$, $^{\#\#\#}p<0.001$).

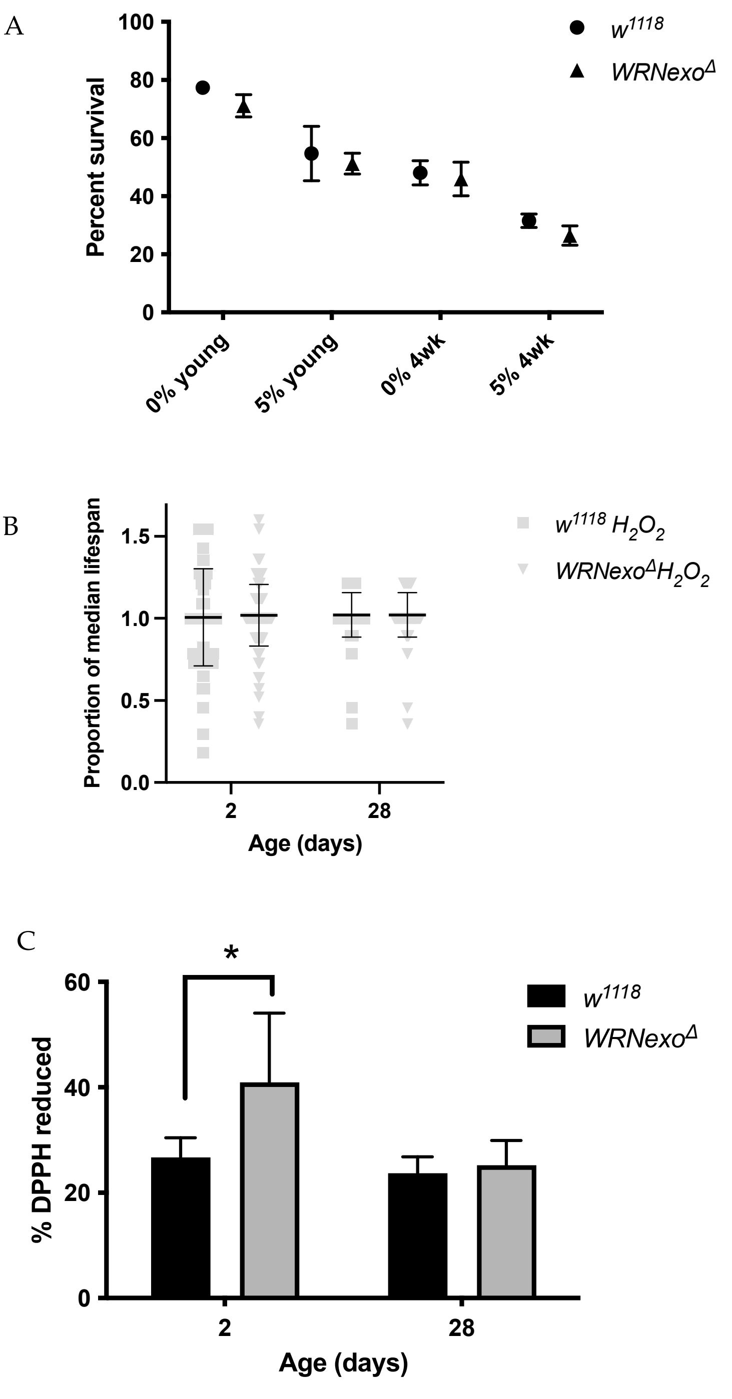

Figure S4: Adult male *WRNexo*$^{\Delta}$ are not sensitive to hydrogen peroxide at 2 and 28 days, but do show high antioxidant activity in young flies. A) Summary data depicting mean lifespan of males exposed to hydrogen peroxide. (2-way ANOVA, p>0.05, ± SEM, n=2-3 independent experimental replicates/conditions. B) Normalized lifespans following 5% hydrogen peroxide showed resistance in *WRNexo*$^{\Delta}$ males to hydrogen peroxide was similar to females (2-way ANOVA, $*p>0.05$, ± SD, n = 157-235 males from 2-3 biological replicates). C) Crude protein extracts from young *WRNexo*$^{\Delta}$ adults had the greatest neutralization effect on the stable free radical DPPH demonstrating higher antioxidant activity ($*p<0.05$ by paired t-test)

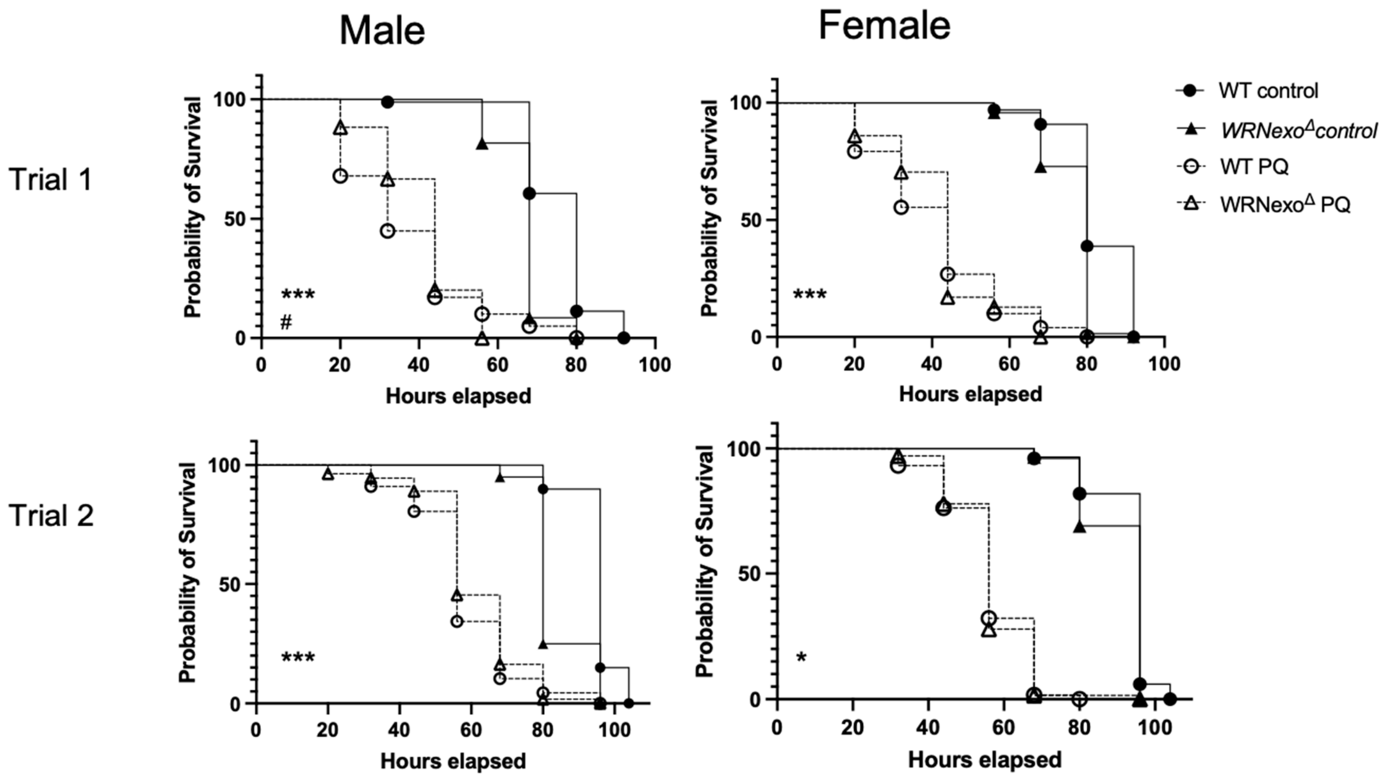

Figure S5: Kaplan-Meyer survival curves for young adult *WRNexo*$^{\Delta}$ in response to paraquat. Normalized lifespans following 20 mM paraquat (PQ) showed sensitivity in *WRNexo*$^{\Delta}$ males, but no sensitvity in *WRNexo*$^{\Delta}$ females (n = 2 biological replicates containing 50-96 flies, ± SD, Mantel-Cox log-rank between *w*^{1118} and *WRNexo*$^{\Delta}$ controls: *p<0.05, ***p<0.001; Mantel-Cox log-rank between H_2O_2-treated *w*^{1118} and *WRNexo*$^{\Delta}$: $^{\#}p$<0.05).2-way ANOVA, *p<0.05, ***p<0.001, ****p<0.0001).

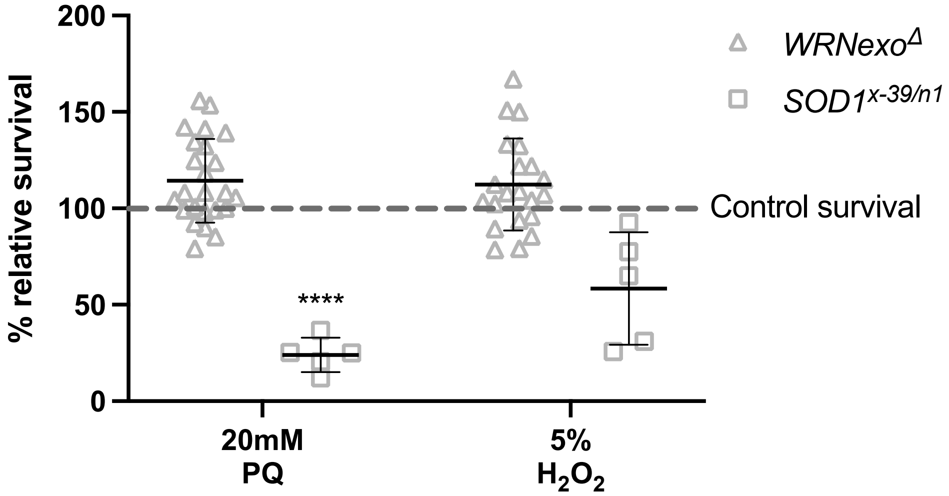

Figure S6: **Oxidative stress does not impact survival of *WRNexo*$^{\Delta}$ larvae.** *WRNexo*$^{\Delta}$ larvae were treated with oxidative stressors 5% hydrogen peroxide (H_2O_2) or 20 mM paraquat (PQ). Superoxide mutant flies (*SOD1*$^{X-39}$/*SOD1*^{n1}) were used as a positive control for PQ sensitivity. Adult survival was determined by dividing the percentage of treated homozygous adults by the percentage of control homozygous adults in each vial (n= 5-24 vials each containing > 20 flies). Neither of these stressors tested caused *WRNexo*$^{\Delta}$ survival to decrease significantly from 100% survival (Student's t-test, ****p<0.001, ± SD).

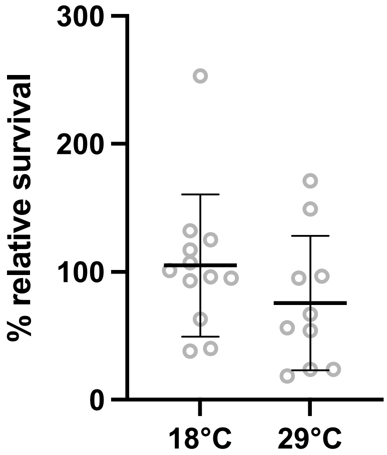

Figure S7: **Non-optimal ambient temperature does not impact survival of *WRNexo*^{Δ} larvae.** *WRNexo*^{Δ} larvae were treated low (18C) and elevated (29C) ambient temperature to induce stress. Adult survival was determined by dividing the percentage of treated adults by the percentage of control adults in each vial (n= 10-12 vials each containing > 20 flies). Non-optimal ambient temperature did not cause *WRNexo*^{Δ} survival to decrease significantly from 100% survival (Student's t-test, p>0.05, ± SD).

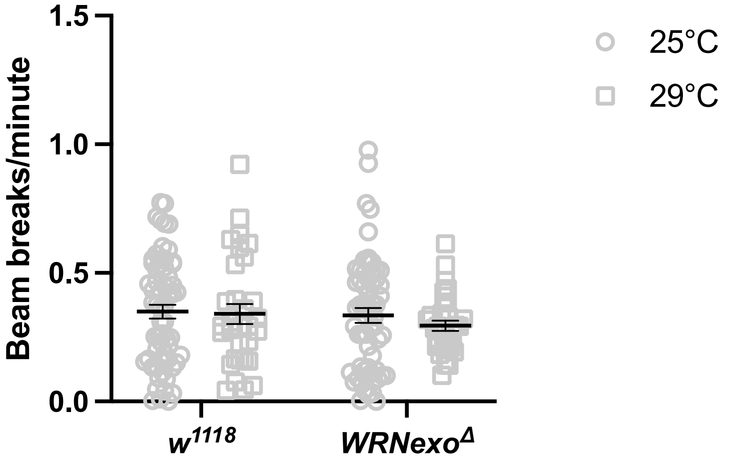

Figure S8: Elevated ambient temperature does not impact overall activity of *WRNexo*$^{\Delta}$ males. Drosophila activity monitor data were collected for young (1–4 day old) w^{1118} and *WRNexo*$^{\Delta}$ males over a 6-day period. Flies were maintained at elevated ambient temperature (29C) to induce stress. Overall *WRNexo*$^{\Delta}$ male activity was not impacted by thermal stress (Kruskal-Wallis test, $p>0.05$).

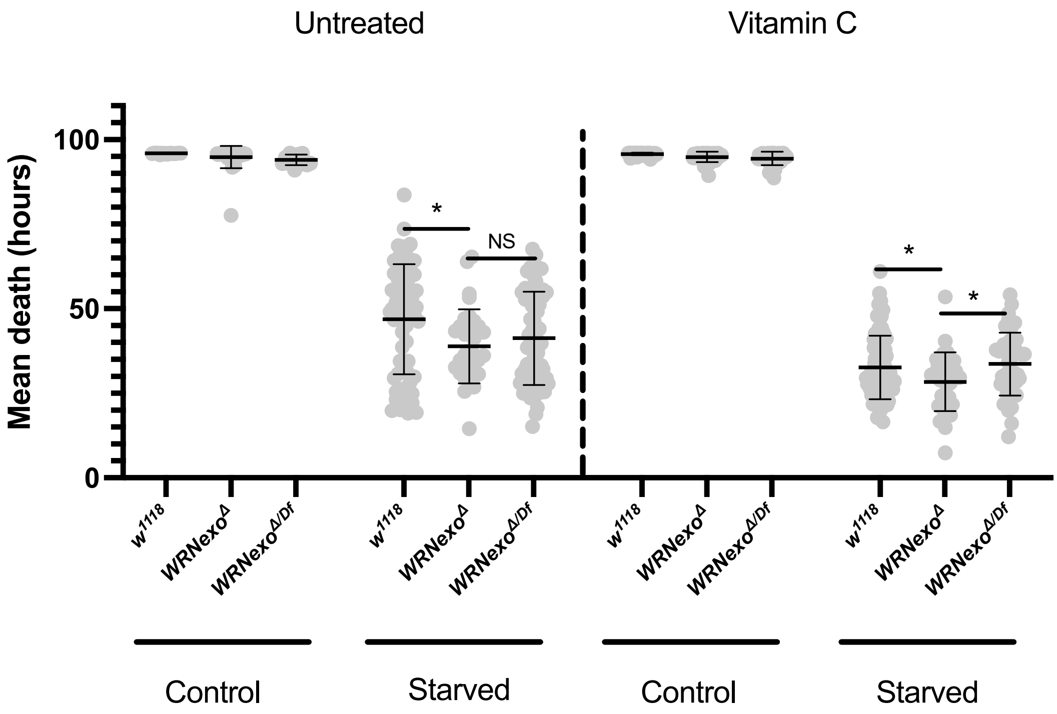

Figure S9: *WRNexo$^{\Delta}$* males show similar starvation sensitivity as *WRNexo$^{\Delta}$* females that is not impacted by vitamin C treatment. *WRNexo* males have shorter mean lifespans under starvation regardless of vitamin C treatment (2-way ANOVA and Tukey's multiple comparisons post-hoc test, *p<0.05, ± SD, n = 16-80 flies/genotype). Df = deficiency strain: *WRNexo$^{\Delta/Df509}$* for untreated experiments and *WRNexo$^{\Delta/Df6178}$* for experiments with vitamin C treatment.

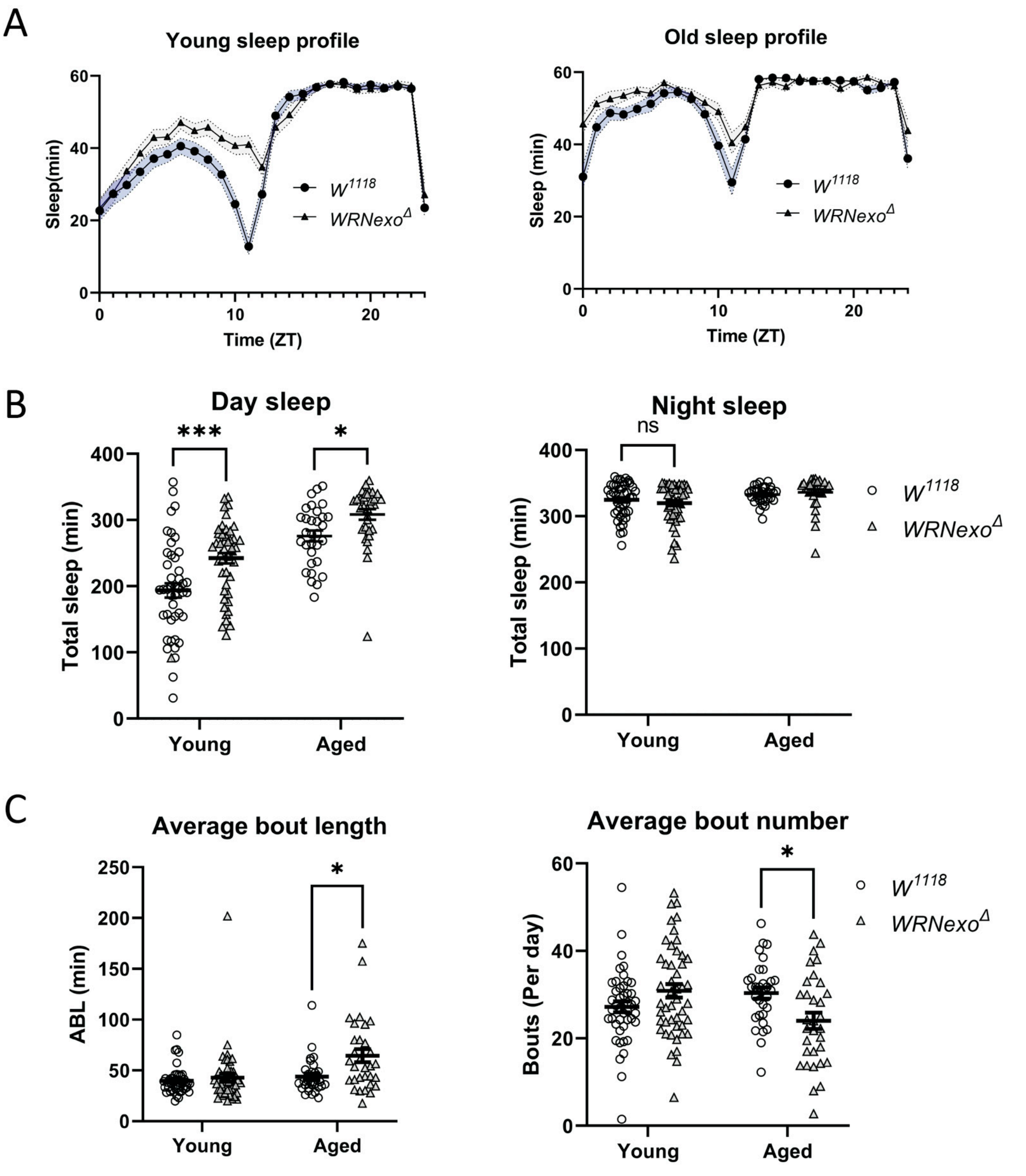

Figure S10: Sleep profile in *WRNexo*$^{\Delta}$ males. A) w^{1118} and *WRNexo*$^{\Delta}$ males exhibit similar levels of sleep throughout the day regardless of age. B) *WRNexo*$^{\Delta}$ males exhibit increased sleep during the day, but no difference in sleep at night compared to w^{1118}. C) Aged *WRNexo*$^{\Delta}$ males show fewer sleep bouts with greater length. (2-way ANOVA, *p<0.05, ***p<0.001, ****p<0.0001, ± SEM).
